# Internet Use Related to Suicidal Thoughts and Help-Seeking – Preliminary Results of a Study with Depressive Patients

**DOI:** 10.1192/j.eurpsy.2022.2163

**Published:** 2022-09-01

**Authors:** V. Voros, Z. Szabo, E. Torma, A. Nagy, J. Fekete, T. Tényi, S. Fekete, P. Osvath

**Affiliations:** University of Pécs, Department Of Psychiatry And Psychotherapy, Pécs, Hungary

**Keywords:** suicidal thoughts, help-seeking, Internet use, suicide prevention

## Abstract

**Introduction:**

Suicide-related Internet use is becoming more common, and many research study its potential risks and benefits.

**Objectives:**

Data on suicidal thoughts and Internet use of patients with depressive disorders were collected to assess their suicide-related Internet use and its relation to help-seeking preferences.

**Methods:**

Semi-structured interviews were completed to assess Internet use patterns and suicidal thoughts among patients treated with depressive disorders, and preferred forms of help-seeking were also examined.

**Results:**

113 patients completed the interviews, most of them spend more hours a day using the Internet. More than 80% came across suicide-related contents while browsing, a quarter reported specific search for suicidal contents. In case of suicidal thoughts, three-quarters of depressed patients would seek help from a mental health professional, two-thirds from their partners, half from friends, and nearly one-third from parents, other relatives or from GPs. Most patients would prefer offline (personal) help for their psychological problems and suicidal thoughts, online methods were less preferred, with only one-fifth choosing these options. However, a third of them also considered it probable that they would not ask anyone for help.

**Conclusions:**

Despite of the frequent use of the Internet, online help-seeking is less common in depressive patients. However, in the times of pandemic, online help facilities may provide an opportunity to prevent suicidal behavior for those, who have suicidal thoughts and use the Internet regularly, especially searching for suicide-related contents. In the future, further research is needed to develop more effective online suicide prevention programs and applications.

**Disclosure:**

No significant relationships.

